# Acute effect of high-definition and conventional tDCS on exercise performance and psychophysiological responses in endurance athletes: a randomized controlled trial

**DOI:** 10.1038/s41598-021-92670-6

**Published:** 2021-07-06

**Authors:** Daniel Gomes da Silva Machado, Marom Bikson, Abhishek Datta, Egas Caparelli-Dáquer, Gozde Unal, Abrahão F. Baptista, Edilson Serpeloni Cyrino, Li Min Li, Edgard Morya, Alexandre Moreira, Alexandre Hideki Okano

**Affiliations:** 1 Associate Graduate Program in Physical Education - UEM/UEL, State University of Londrina, Londrina, PR Brazil; 2 Department of Physical Education, Universidade Federal do Rio Grande do Norte, Natal, RN Brazil; 3 Department of Biomedical Engineering, The City College of New York of CUNY, New York, NY USA; 4 Nervous System Electric Stimulation Lab (LabEEL), Rio de Janeiro State University (UERJ), Rio de Janeiro, RJ Brazil; 5 Center of Mathematics, Computation, and Cognition, Universidade Federal do ABC, São Bernardo do Campo, SP Brazil; 6 Brazilian Institute of Neuroscience and Neurotechnology (BRAINN/CEPID-FAPESP), Faculty of Medical Sciences, Department of Neurology, University of Campinas, Campinas, São Paulo Brazil; 7 Edmond and Lily Safra International Institute of Neuroscience, Santos Dumont Institute, Macaíba, RN Brazil; 8 Department of Sport, School of Physical Education and Sport, University of São Paulo, São Paulo, SP Brazil

**Keywords:** Motor cortex, Neurophysiology, Motivation

## Abstract

Transcranial direct current stimulation (tDCS) has been used aiming to boost exercise performance and inconsistent findings have been reported. One possible explanation is related to the limitations of the so-called “conventional” tDCS, which uses large rectangular electrodes, resulting in a diffuse electric field. A new tDCS technique called high-definition tDCS (HD-tDCS) has been recently developed. HD-tDCS uses small ring electrodes and produces improved focality and greater magnitude of its aftereffects. This study tested whether HD-tDCS would improve exercise performance to a greater extent than conventional tDCS. Twelve endurance athletes (29.4 ± 7.3 years; 60.15 ± 5.09 ml kg^−1^ min^−1^) were enrolled in this single-center, randomized, crossover, and sham-controlled trial. To test reliability, participants performed two time to exhaustion (TTE) tests (control conditions) on a cycle simulator with 80% of peak power until volitional exhaustion. Next, they randomly received HD-tDCS (2.4 mA), conventional (2.0 mA), or active sham tDCS (2.0 mA) over the motor cortex for 20-min before performing the TTE test. TTE, heart rate (HR), associative thoughts, peripheral (lower limbs), and whole-body ratings of perceived exertion (RPE) were recorded every minute. Outcome measures were reliable. There was no difference in TTE between HD-tDCS (853.1 ± 288.6 s), simulated conventional (827.8 ± 278.7 s), sham (794.3 ± 271.2 s), or control conditions (TTE1 = 751.1 ± 261.6 s or TTE2 = 770.8 ± 250.6 s) [F_(1.95; 21.4)_ = 1.537; *P* = 0.24; η^2^p = 0.123]. There was no effect on peripheral or whole-body RPE and associative thoughts (*P* > 0.05). No serious adverse effect was reported. A single session of neither HD-tDCS nor conventional tDCS changed exercise performance and psychophysiological responses in athletes, suggesting that a ceiling effect may exist.

## Introduction

Fatigue, defined as an exercise-induced decrease in the ability to generate force or power, can be related not only to the failure in contractile properties of the muscle but also by a limited descending command from the nervous system (i.e. neural drive)^1^. During fatiguing exercise, there is a progressive increase in the descending outputs (i.e. neural drive) from the primary motor cortex (M1) to counteract the decrease in spinal motoneurons excitability^1–3^. The failure to increase M1 outputs to the motor neurons would contribute to fatigue^2–4^. The involvement of different brain regions has also been proposed to influence exercise perception and performance such as the dorsolateral prefrontal cortex (DLPFC), primary sensory cortex, supplementary motor area, insular cortex^5–8^. This highlights the importance of the central nervous system for exercise perception and performance and also suggests that interventions that could modulate its activity could impact exercise perception and performance.

Transcranial direct current stimulation (tDCS) consists of applying a weak electrical current (up to 4 mA) on the scalp over a brain region of interest^9^ that can modulate neuronal excitability and this effect outlasts the stimulation period for several minutes^10,11^. tDCS is a relatively inexpensive neuromodulatory technique, portable, and easy to use, which makes an interesting alternative for the sporting context. The neuronal modulation from tDCS could affect the central fatigue related to exercise, for instance, by increasing M1 excitability and possibly the neural drive, which in turn, could possibly postpone fatigue and improve exercise performance^5,12,13^. In fact, tDCS has shown promising results in the sports and exercise science field^5,14–19^. For example, studies have shown that anodal tDCS (a-tDCS) applied over M1 improved endurance performance in cycling in physically active (non-athletes) individuals^17,18^. Furthermore, Okano et al.^16^ were one of the firsts to demonstrate the modulation of whole-body exercise performance and exercise-related perception by tDCS. They showed that a-tDCS (2 mA for 20 min) applied over the temporal cortex (TC), targeting the left insular cortex, improved cycling performance by approximately 4% in professional cyclists and also decreased heart rate (HR) and ratings of perceived exertion (RPE) in submaximal intensities^16^. Others have also found decreased RPE during endurance cycling test^18,20^, dynamic resistance exercise^21,22^, isometric exercise^23^ with tDCS targeting M1 and DLPFC. On the other hand, no effect of tDCS targeting either M1, TC, or DLPFC on RPE was found in cycling^24–27^, running^28^, and swimming^29^. Interestingly, improved exercise performance has been reported even in the absence of changes in RPE^17,30^. Besides, meta-analytical evidence has shown improvements in muscle strength and endurance during an exercise involving isometric and dynamic contractions^19^.

The interest in tDCS, supported by the findings of these studies, has crossed the laboratories walls, so that a commercial company claimed, in an unpublished data, that tDCS applied over M1 improved jumping force and coordination of American Olympic ski jumpers by 70% and 80%, respectively^31^ and there is also a report of tDCS use for performance enhancement in athletes from one of the NBA’s top-teams^32^. However, only studies with physically active individuals (non-athletes) have been published in peer-reviewed journals showing a positive effect on flexibility, lower limb power, sprint cycling, and endurance running^30,33,34^. Furthermore, even though most reports advocate in favor of tDCS-induced performance enhancement only a handful of studies were conducted with actual athletes involving different motor tasks such as motor slowing in hand- and foot-tapping tasks, time-trial, and incremental tests in cycling, running, and swimming^16,26,29,35,36^. Moreover, several studies did not found performance improvements^24–26,29,37,38^.

In this regard, Machado et al.^5^ performed a systematic review with meta-analysis including 22 studies and found that M1 was the main nominal target for a-tDCS and also found weak evidence that a-tDCS over M1 improved cycling performance by ~ 93 s (95% CI = 27.39–159.43 s). However, this significant effect was strongly influenced by a single study. Another meta-analysis showed a positive effect on performance enhancement and subjective measures of exercise (i.e., RPE), however, the authors included studies applying a-tDCS over multiple regions (M1, DLPFC, and TC) and/or included measures of muscular strength, endurance, and whole-body exercise performance in the same meta-analysis^15,19^.

The inconsistent results presented in the literature might be explained by the variations in the tDCS technique among studies. For instance, the position of the return electrode (cathodal for studies using anodal as the intervention) differs among studies, with some studies positioning on the contralateral orbitofrontal area, DLPFC, inion, or shoulder^5^, which neglects the role of the return electrode^39,40^. Furthermore, the duration of the intervention and current density also varies among studies ranging from 10 to 20 min and from 0.056 to 0.44 mA/cm^2^, respectively^5^, which may change the amount of current that is actually delivered to the brain. Moreover, and likely the most important, the majority of these studies did not use any kind of measure to predict the appropriate electrode montage (i.e., electrode size, position, and current intensity) for ensuring that the nominal target would actually be stimulated, such as computational modeling^5,39,41^.

The most recognized characteristic of the so-called “conventional” tDCS, in which the electrical current is applied through large rectangular electrodes pads of conductive material, is that the current path is diffuse and the peak electric field is not strictly under the electrode (i.e. low focality), as it is commonly assumed^39–41^. To overcome this limitation, a new form of tDCS application, called “high-definition” tDCS (HD-tDCS), was recently developed^42^. With HD-tDCS the electrical current is applied through ring electrodes and is circumscribed to the ring diameter^42^. Studies have shown that HD-tDCS, compared to conventional tDCS, present greater focality, stimulating with a gyri-level precision^42–45^, a greater magnitude of neuronal excitability change^10^, longer duration of its after-effects^10^, and also better-developed sham methods^45^. Thus, it would be reasonable to speculate that HD-tDCS could present greater effects on behavioral outcomes compared to conventional tDCS. So far, however, it remains speculative as only three studies used HD-tDCS^37,38,46^. Two of them for investigating exercise performance with low-intensity isometric contraction (30–35% of maximum isometric voluntary contraction) performed to exhaustion and found no change in performance^37,38^. In a recent study, Pollastri et al.^46^ assessed the effect of a bilateral HD-tDCS over the DLPFC (F3 and F4) for 20 min on a 15 km time-trial performance in eight elite cyclists. The authors reported that the time to complete the time trial was 1.3% faster after HD-tDCS^46^. However, none of these studies compared HD-tDCS to conventional tDCS. However, it is not clear whether the increased focality and greater excitability change in the motor cortex found with motor evoked potential using HD-tDCS would translate into greater exercise performance compared to conventional tDCS^10^. Therefore, this study aimed to compare the effects of HD-tDCS to conventional tDCS applied over M1 on whole-body exercise performance and psychophysiological responses in endurance athletes. We hypothesized that both conventional and HD-tDCS would improve exercise performance, with a greater effect of the latter due to its higher precision, greater magnitude^10^, and duration of the modulation of corticospinal excitability^10,42–45^. It was also hypothesized based on previous findings that both tDCS techniques would decrease psychophysiological responses at submaximal loads^16,30^.

## Methods

### Study design

**Figure 1:**
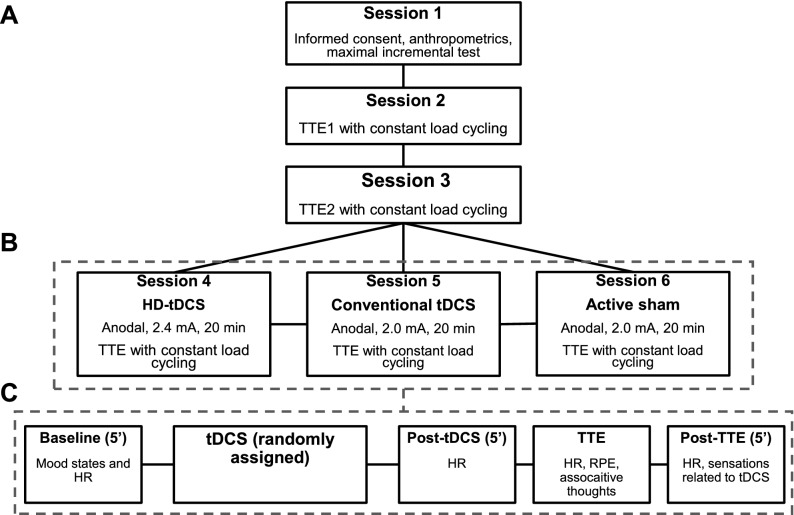
Flowchart of the study. Incremental test and reproducibility of the outcome measures (**A**), experimental phase (**B**), detailed experimental phase sessions (**C**). Note: the order of the experimental sessions was randomized, the numbers in the boxes do not imply a fixed order. HD-tDCS = high-definition transcranial direct current stimulation; HR = heart rate; RPE = ratings of perceived exertion; tDCS = transcranial direct current stimulation; TTE = time to exhaustion.

This was a single-center, randomized, crossover, single-blinded, sham-controlled trial with a within-subject design to compare the effects of HD-tDCS and conventional tDCS on exercise performance. This study was divided into two phases: phase I (reliability phase, aiming to assess the reliability of the outcome measures) and phase II (experimental phase, aiming to test the effect of tDCS). Participants had to participate in six sessions at the exercise science laboratory at the University. In the first session, participants received information regarding the study procedures and, after signing an informed consent form, they underwent an anthropometric assessment and performed a maximal incremental test on a cycle simulator. In the next two sessions, they performed a constant load time to exhaustion test (TTE) on a cycle simulator, without any intervention, to test the reproducibility of their performance, physiological, and psychophysiological measures (these sessions were not included in the randomization). In the subsequent three sessions, participants received either anodal HD-tDCS, anodal conventional tDCS, or active sham tDCS for 20 min, in a randomized order, before performing the TTE test. There was a minimal and maximal interval of 48 h and one week between sessions, respectively. The order of the tDCS sessions was randomized. The randomization sequence was generated for 15 participants by a computer (http://www.randomization.com/) using random balanced permutations. Participants were allocated to a predefined sequence according to their enrollment so that the sequence of the tDCS sessions did not depend on the researchers. The study design is presented in Fig. 1. The study protocol was registered on the Brazilian Registry of Clinical Trials (Registration Date: 07/08/2020), under the register number RBR-4jvybh (http://www.ensaiosclinicos.gov.br/rg/RBR-4jvybh/). The data acquisition occurred from November 2017 to January 2018. This study was approved by the Institutional Ethics Committee of the Federal University of Rio Grande do Norte (CAAE: 73743317.6.0000.5537; protocol number: 2.363.003) and was conducted following the Declaration of Helsinki. All participants gave their written informed consent. This study was reported following the CONSORT guidelines^47^.

### Participants

The sample of this study was composed of healthy male athletes aged from 19 to 41 years, who had participated in competitions of regional, national, and international levels on the current season and/or during their sports career. Twenty-two participants entered the study, and 16 participants completed phase I (reproducibility), ten cyclists, and six rowers (six participants did not complete phase I due to time constraints or gave no reason/no response). Twelve participants completed the entire experiment, seven cyclists and five rowers. Four participants did not complete the study due to time constraints. All participants' characteristics are presented in Table 1. The sample size calculation, for the experimental phase, was performed a priori based on the results of a previous study^17^ for a repeated-measures ANOVA with a within-subjects design using effect size (partial eta squared) = 0.45, α = 0.05, power = 0.80, number of groups = 1, number of measurements = 3. Accordingly, at least nine subjects would be necessary for this study. The sample size calculation was performed using Gpower software v.3.1.9.2 (Universität Kiel, Kiel, Germany) with the option “as in SPSS”. The effect size of Vitor-Costa et al.^17^ was reduced considering that we would enroll athletes, which could be expected an effect of smaller magnitude.

**Table 1:** General characteristics of the sample.

Variables	Reproducibility phase (n = 16)	Experimental phase (n = 12)
Age (years)	30.3 ± 8.2	29.4 ± 7.3
Body mass (kg)	72.8 ± 9.2	72.0 ± 7.9
Height (m)	1.74 ± 0.05	1.74 ± 0.06
Body mass index (kg/m^2^)	24.01 ± 2.77	23.75 ± 2.29
Fat-free mass (kg)	56.16 ± 5.74	56.13 ± 5.52
Fat mass (kg)	14.02 ± 5.19	13.27 ± 3.67
Body fat (%)	19.49 ± 5.19	18.93 ± 3.92
Training sessions (days/week)	4.8 ± 1.68	4.9 ± 1.7
Training duration (min/day)	123.8 ± 42.2	125.0 ± 35.8
Training experience (years)	8.12 ± 6.85	6.87 ± 4.88
Resting heart rate (bpm)	48.6 ± 5.5	47.4 ± 3.8
Resting VO_2_ (ml kg^−1^ min^−1^)	4.13 ± 0.98	4.00 ± 0.62
Maximum heart rate (bpm)	187.0 ± 8.3	185.6 ± 7.6
Maximum VO_2_ (ml kg^−1^ min^−1^)	60.13 ± 4.91	60.15 ± 5.09
Peak power output (W)	340.1 ± 53.2	334.6 ± 53.0
80% of peak power output (W)	272.0 ± 42.6	267.6 ± 42.4

Note: data are presented as mean ± standard deviation; VO_2_ = oxygen uptake.

Individuals were recruited via personal invitation and fliers in groups of athletes on social media. To be included in this study individuals had to: (a) aged between 18 and 50 years; (b) take part in physical training with competitive purposes during the last six months; (c) participate in endurance competitions; (d) free from any neurological or psychiatric disorder; (e) not taking any medication that could affect the central nervous system; (f) no contraindication for tDCS (i.e. not having metal implanted in the head, pacemaker, medical bumps, seizures, lesions on the scalp or head).

### Anthropometrics and body composition assessment

Body mass (kg) and height were measured in the first session using an electronic scale with a stadiometer (Welmy, W110H, Santa Bárbara d´Oeste, SP, Brazil) with the participant wearing light clothes (appropriate for exercising) using standard procedures. Body mass index was calculated as the ratio between body mass and height squared. Also, a dual-energy X-ray absorptiometry (DEXA; Lunar Prodigy, GE Medical System, Madison, WI, USA) scan was used to assess body composition (i.e. body fat, fat-free mass). The intraclass correlation coefficient (ICC), standard error of measurement (SEM), and minimal detectable change (MDC) for these variables were as follows: body mass (ICC = 1.00; SEM = 0.000; MDC = 0.0; CV = 15.8); height (ICC = 0.999; SEM = 0.002; MDC = 0.14; CV = 4.47); fat free mass (ICC = 0.998; SEM = 0.384; MDC = 1.718; CV = 14.9); bone mass (ICC = 0.992; SEM = 0.471; MDC = 1.902; CV = 27.2); and percentage of body fat (ICC = 0.992; SEM = 0.471; MDC = 1.902; CV = 27.2).

### Maximal incremental test

The maximal incremental test was performed on a cycle simulator (Velotron Dynafit Pro, RacerMate, Seatle, USA). Before the test, the participants adjusted the cycle simulator according to their preference and the settings (seat and handlebar heights and distance) were recorded to be reproduced in the remaining sessions. The test began with 100 W for two minutes and increments of 50 W every two minutes. HR and respiratory gas exchange were continuously measured, and participants were asked to report RPE and associative thoughts during the last 15 s of each stage. Participants cycled at preferred cadence, with a lower limit of 60 rpm. The test ended when the individual could not maintain cycling cadence over 60 rpm (> 5 s) or due to volitional exhaustion. Peak power output (PPO) was considered as the sum of the power in the last completed stage plus the product of the percent time spent in the stage in which exhaustion occurred.

### Heart rate and ventilatory measures

HR was recorded continuously, during all sessions, using an HR monitor (RS800cx, Polar Electro OR, Finland) with a sampling rate of 1000 Hz. The HR data were downloaded by Polar Pro Trainer 5 (Polar, Finland) for further analysis. The respiratory gas exchange was continuously analyzed during the incremental test and TTE tests during the reproducibility phase using a metabolic cart (Quark CPET, Cosmed, Rome, Italy) on a breath-by-breath basis. Before each test, the equipment was calibrated following the manufacturer’s instructions and using a gas mixture of known concentration and a 3-L calibration syringe (Cosmed, Rome, Italy). For the analysis, the data were averaged by 15 s, and the last value for each minute of the test was used for the analysis. Also, the highest 15 s averaged O_2_ uptake value obtained during the incremental test was defined as the maximum oxygen uptake (VO_2max_). Resting HR and oxygen uptake was measured before the incremental test, with the individual seated and relaxed for five minutes.

### Transcranial direct current stimulation intervention

**Figure 2:**
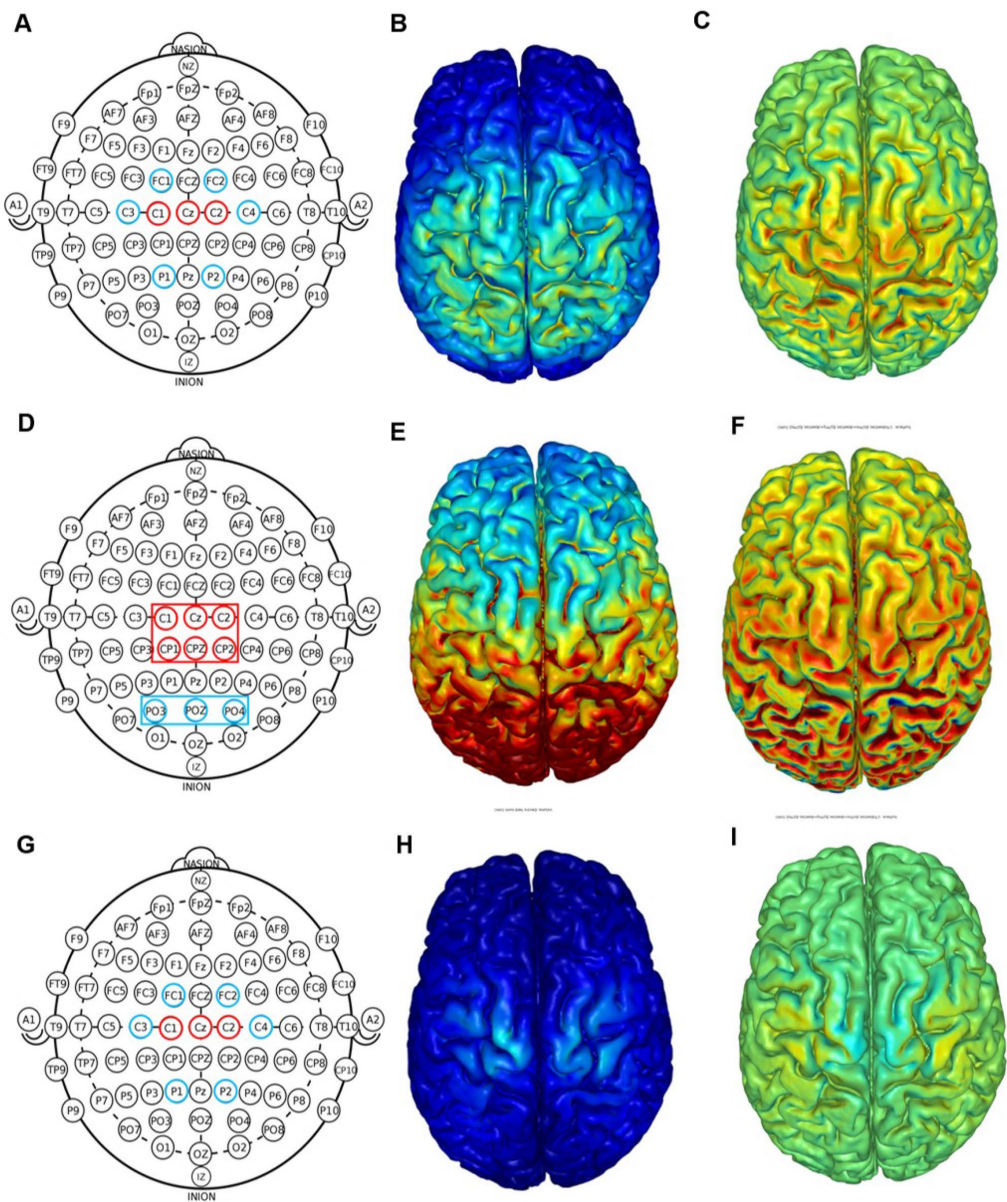
Transcranial direct current stimulation montages used in the present study: high-definition (top row), simulated conventional (middle row), and active sham (bottom row). Ring electrode positions (red = anodal electrode; blue = cathodal electrode) according to the EEG 10/20 system (**A**, **D**, **G**). For the conventional montage, ring electrodes were used to simulate the rectangular electrodes (**D**). Computational modeling of the finite element model of the total (**B**, **E**, and **H**) and radial (**C**, **F**, and **I**) electric fields induced by high-definition and active sham tDCS. All figures are displayed with a field intensity of 0.30 V/m (directionless. blue = zero electric fields; red = peak magnitude). Radial electric field (**C**, **F**, and **I**) considers the direction of current (blue = peak outward current; green = zero normal current; red = peak inward current). Note: panels B, C, E, F, H, and I was generated by the HD-Explore software (Version 2.3, Soterix Medical, New York, NY; https://soterixmedical.com/research/software/hd-explore).

Before the constant load TTE test, individuals received either anodal HD-tDCS, anodal simulated conventional tDCS, or sham tDCS for 20 min. Participants were blinded to the tDCS condition they were receiving. tDCS electrodes were fixated into plastic casings appropriate for concomitant EEG-tDCS measurements, which was attached to an EEG cap, with 64 channel positions according to the 10/20 EEG international system, adequate for individuals' head size (Acticap; Brain Products, Munich, Germany). Each plastic casing was filled with approximately 2 ml of HD-tDCS gel (Soterix Medical, New York, NY) to make contact between the electrode and the scalp. tDCS was started only after the impedance was < 30 kOhms. Nine ring Ag–AgCl electrodes connected to a tDCS device (MxN, Soterix Medical, New York, NY) were used for all tDCS conditions. For all tDCS conditions, the current was gradually increased and decreased in the first and last 30 s. All tDCS montages were determined based on computational modeling using a finite element model of the brain current flow during tDCS. HD-Explore software (Version 2.3, Soterix Medical, New York, NY) was used to determine electrode location, current intensity, as well as tDCS, induced electrical field (Fig. 2).

For HD-tDCS, a current intensity of 2.4 mA was applied for 20 min with the following electrode location and current: C3 (− 0.4 mA), C4 (− 0.4 mA), Cz (0.8 mA), FC1 (− 0.4 mA), FC2 (− 0.4 mA), C1 (0.8 mA), C2 (0.8 mA) P1 (− 0.4 mA), P2 (− 0.4 mA). The montage was planned so that the highest electrical field would be induced in the motor representation of the lower limbs (Fig. 2A-C). The conventional tDCS montage was based on the montage used by Vitor-Costa 17 with rectangular pad electrodes with the anode (36 cm2 ) over the motor representation of the lower limbs (Cz) and the return electrode (35 cm2 ) over the occipital protuberance (inion). Conventional tDCS was simulated with HD-tDCS electrodes to avoid participant awareness regarding the difference in the tDCS application form (see Fig. 2D for comparison). The current intensity was set at 2.0 mA for 20 min. Electrode location and current intensity were set as follows (Fig. 2D–F): C1, Cz, C2, CP1, CPz, CP2 (0.33 mA each), and PO3, POz, PO4 (− 0.66 mA each). The computational modeling showed that the simulated montage induced the same amount of radial electric field as the one used by the quoted study with rectangular pad electrodes^17^.

An active sham was applied with 2.0 mA for 20 min with the same electrode location as the HD-tDCS condition, but with different current intensity for each electrode as follows: C3 (− 0.1 mA), C4 (− 0.1 mA), Cz (− 1.2 mA), FC1 (− 0.2 mA), FC2 (− 0.2 mA), C1 (1.0 mA), C2 (1.0 mA) P1 (− 0.1 mA), P2 (− 0.1 mA) (Fig. 2G–I). The adequacy of the ramp-up/down has been debated, and its suitability will depend on the overall experimental design. Our use of an “active” sham has been used by others^48–50^, providing a more reliable reproduction of stimulation sensations (i.e., scalp current stimulation for the entire duration but with a minimal transcranial current flow). Moreover, we develop a system where comparable head-gear across conditions supports blinding.

At the end of each session, participants filled a questionnaire proposed by Fertonani et al.^51^ indicating the sensations and the degree of intensity felt during the stimulation. The questionnaire included the following sensations: itching, pain, burning, warmth/heat, pitching metallic/iron taste, fatigue, others (opened). The degrees were none (0), mild (1), moderate (2), considerable (3), strong (4). Participants also reported when the discomfort began (1 = beginning, 2 = at approximately the middle, 3 = towards the end),how long it lasted (1 = stopped quickly; 2 = stopped in the middle; 3 = stopped at the end), and if these sensations affected their exercise performance (0 = not at all; 1 = slightly; 2 = considerably; 3 = much; 4 = very much).

### Mood states assessment

To control for psychological state variations between tDCS conditions that could influence exercise performance, the mood states were evaluated before each study session using the Brunel Mood Scale, which is a 24-item self-reported measure divided into six subscales: anger, confusion, depression, fatigue, tension, and vigor. Participants rated their mood state on a 5-point Likert scale from 0 (not at all) to 4 (extremely), based on how they were feeling at the moment of evaluation.

### Time to exhaustion test with constant load

Participants performed a test with constant load fixed at 80% of PPO in the same cycle simulator as the incremental test (Velotron Dynafit Pro, RacerMate, Seatle, USA) and were instructed that the purpose of the testing was to cycle for as long as possible (i.e., until exhaustion). Participants cycled at preferred cadence, with a lower limit of 60 rpm. The test ended when the individual could not maintain cycling cadence over 60 rpm (> 5 s) or due to volitional exhaustion. Strong verbal encouragement was provided during the entire test. The TTE was defined as the time elapsed between the beginning of the test and the very last second in which cadence was ≥ 60 rpm. This was precisely measured as the cycle simulator registers in file information regarding cadence, speed, and the distance covered every 30 ms. During the TTE test, HR was continuously measured, and participants reported RPE and associative thoughts in the last 15 s of each minute. Participants were not informed of the duration of their session. Only after the last session, they were informed of their times. Figure 1C summarizes the flow of the experimental sessions.

### Perceived exertion and associative thoughts

RPE was measured using the Borg 6–20 RPE scale to estimate peripheral (lower limbs) and whole-body perceived exertion during exercise. RPE anchoring was number 9 represents very light exercise intensity while number 19 indicates an exertion similar to exhaustive cycling^52^. RPE was defined to participants as the intensity of effort and exertion felt during the exercise^52^.

Associative thoughts were measured as a proxy of the attentional focus and were defined as those directed towards bodily symptoms and measured using a scale ranging from zero (0) to 100%, where 100 represents only associative thoughts. Dissociative thoughts were defined as thoughts that distract the subjects from the exercise being performed and directed towards external factors and were measured as the remainder of the thought score not described as associative thoughts^53^. Standard instructions about reporting RPE and associative thoughts were given before the maximal incremental session and remembered before starting each TTE test. Participants reported RPE and associative thoughts in the last 15 s of each minute. The scales were presented in a random order to avoid automated responses.

### Statistical analysis

The normal distribution of the data was analyzed by Shapiro–Wilk’s test. Accordingly, descriptive statistics were used to describe sample characteristics with either mean and standard deviations or median and interquartile range.

#### Reproducibility phase

The paired t-test was used to compare TTE, HRmax, VO_2_max, and Wilcoxon test to compare baseline psychological state. In addition, the intraclass correlation coefficient (ICC) was used to assess the test–retest reliability of the two best performances. Also, the standard error of measurement [SEM = SD × √(1 − r)] and the minimum detectable change [MDC = 1.96 × √(2 × SEM)] was calculated. Additionally, the agreement between measures was assessed using the Bland–Altman plots, in which the average of the two measurements [(M1 + M2)/2] is displayed on the X-axis and the difference between the two measures (M1–M2) on the Y-axis, for each participant. The agreement was confirmed when the line of equality (zero line) was within the 95% confidence interval (95% CI) of the averaged differences. A two-way analysis of variance (ANOVA) with repeated measures was used to compare the physiological (HR and VO_2_) and psychophysiological (attentional focus, peripheral and whole-body RPE) variables during the TTE test, using the test (TTE1 or TTE2) and the time points as factors for comparisons.

#### Experimental phase

Baseline mood state and the sensations related to tDCS were compared using a Friedman test. A one-way analysis of variance (ANOVA) with repeated measures was used to compare the results of TTE and HR_max_. Furthermore, a two-way ANOVA with repeated measures was used to compare HR, associative thoughts, peripheral and whole-body RPE during TTE using either tDCS condition (HD-tDCS, conventional tDCS, and sham) and the time points as factors for comparisons.

For ANOVAs, the homogeneity of the variances was analyzed using Levene’s test. Mauchly’s test was used to evaluate the sphericity assumption and, whenever sphericity was violated, Greenhouse-Geiser epsilon correction was used. Bonferroni’s post hoc test was used whenever a significant F was found. Partial eta squared (η^2^p) was reported as a measure of the effect size. Also, the effect size was calculated using Hedge’s g for planned comparisons (experimental conditions against sham) with a 95% confidence interval (95% CI). *P* ≤ 0.05 was considered significant. Statistica 8.0 was used for the data analysis.

## Results

### Participants’ characteristics

The general characteristic of the sample for the two phases of the study is presented in Table 1.

### Phase I (reproducibility)

#### Reproducibility of TTE, HR_max_, and VO_2max_

**Figure 3:**
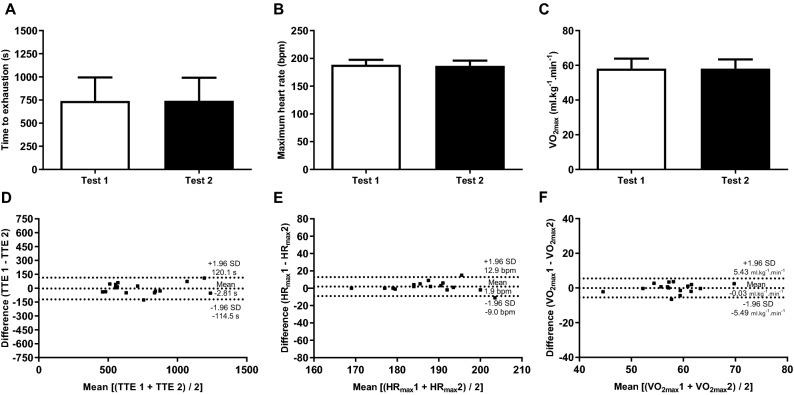
Comparison and Bland–Altman plots between the time to exhaustion (**A** and **D**), maximum heart rate (**B** and **E**), and maximum oxygen uptake (**C** and **F**) of the athletes performance in the reproducibility phase of the study (n = 16).

A paired t-test showed no difference between the two tests in the reproducibility phase for TTE (t = − 0.228; *P* =  0.82; r = 0.97; Fig. 3A), HR_max_ (t = 1.383; *P* =  0.19; Fig. 3B), and VO_2max_ (t = − 0.047; *P* = 0.96; Fig. 3C) attained during TTE test. The TTE presented an excellent reliability with an ICC = 0.987 (95% CI = 0.961–0.995; *P* < 0.0001) and the Bland–Altman plots also confirmed the agreement between measures (Fig. 3D). The SEM and MDC for TTE was 28.2 s and 14.7 s, respectively. In addition, HR_max_ (ICC = 0.894; 95% CI = 0.705 to 0.963; *P* < 0.0001) and VO_2max_ (ICC = 0.938; 95% CI = 0.820 to 0.978; *P* < 0.0001) displayed an excellent reliability and agreement between measures (Fig. 3E–F).

There was no difference in baseline psychological state between the reproducibility sessions: vigor [10.5 (6.25–12.75) vs. 9.0 (6.25–11.5); Z = − 0.992; *P* = 0.32], fatigue [0.0 (0.0–2.0) vs. 0.0 (0.0–1.0); Z = − 0.173; *P* = 0.86], tension [0.0 (0.0–1.75) vs. 0.0 (0.0–1.0); Z = − 1.00; *P* = 0.32], confusion [0.0 (0.0–0.0) vs. 0.0 (0.0–0.0); Z = 0.000; *P* = 1.00], anger [0.0 (0.0–0.0) vs. 0.0 (0.0–0.0); Z = − 1.000; *P* = 0.32], and depression [0.0 (0.0–0.0) vs. 0.0 (0.0–0.0); Z = − 1.000; *P* = 0.32], for the first and second TTE test, respectively.

#### Reproducibility of HR, VO_2_, peripheral RPE, whole-body RPE, and associative thoughts during TTE tests

**Figure 4:**
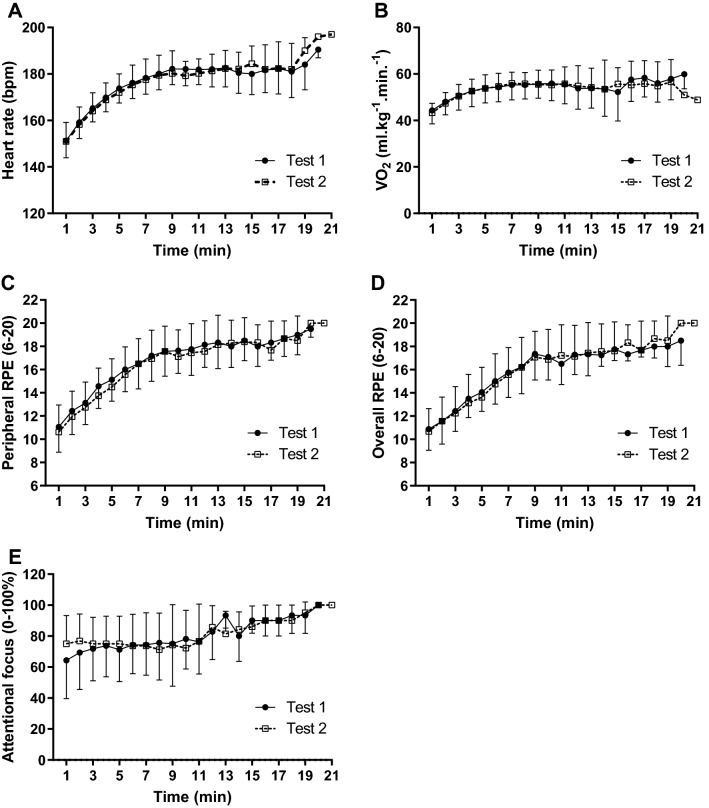
Physiological and psychophysiological responses during the two best performances in time to exhaustion test with 80% of peak power in athletes (n = 16). Heart rate (**A**), oxygen uptake (**B**), perceived exertion of the lower limbs (**C**), perceived exertion of the whole-body (**D**), associative thoughts (**E**), and affective responses (**F**).

Figure 4 displays the physiological and psychophysiological responses obtained during the TTE tests. A main effect of time was found on HR [F_(17, 34)_ = 74.753; *P* < 0.0001; η^2^p = 0.974], with no main effect of test [F_(1, 2)_ = 0.069; *P* = 0.817; η^2^p = 0.033] or test × time interaction [F_(17, 34)_ = 1.381; *P* = 0.206; η^2^p = 0.408] (Fig. 4A). Similarly, a main effect of time was found on VO_2_ [F_(17, 34)_ = 9.767; *P* < 0.0001; η^2^p = 0.830], with no main effect of test [F_(1, 2)_ = 3.262; *P* = 0.213; η^2^p = 0.620] or test × time interaction [F_(17, 34)_ = 0.829; *P* = 0.652; η^2^p = 0.293] (Fig. 4B).

Regarding psychophysiological responses, only a main effect of time was found for peripheral RPE [F_(17, 34)_ = 12.845; *P* < 0.0001; η^2^p = 0.865], with no main effect of test [F_(1, 2)_ = 1.864; *P* = 0.305; η^2^p = 0.482] or test × time interaction [F_(17, 34)_ = 0.257; *P* = 0.998; η^2^p = 0.114] (Fig. 4C). Likewise, a main effect of time was found for whole-body RPE [F_(17, 34)_ = 12.662; P < 0.0001; η^2^p = 0.864], with no main effect of test [F_(1, 2)_ = 0.056; *P* = 0.835; η^2^p = 0.027] or test ×  time interaction [F_(17, 34)_ = 0.498; *P* = 0.936; η^2^p = 0.200] (Fig. 4D). On the associative thoughts, there was no effect of neither test [F_(1, 2)_ = 0.517; *P* = 0.547; η^2^p = 0.205], time [F_(17, 34)_ = 1.355; *P* = 0.220; η^2^p = 0.404], or test × time interaction [F_(17, 34)_ = 1.343; *P* = 0.226; η^2^p = 0.402] (Fig. 4E).

### Phase II (Experimental)

#### Intervention overview

tDCS was well-tolerated with no side or adverse effect being reported. Table 2 summarizes the sensations felt during the stimulation period. There was no difference in sensation type, intensity, and duration between conditions. The most common sensations reported were itching, burning, heating, and pitching that was felt on the head started at the beginning or middle of the stimulation with varying duration. No other sensation was reported. Importantly, all subjects reported these sensations did not affect their performance in any tDCS condition. Regarding the blinding of participants, only one volunteer who participated in a previous study involving conventional tDCS said he was 80% sure that his first session was sham, which was wrong. The rest of the participants believed they were stimulated in all sessions.

**Table 2:** Sensations felt by athletes during high-definition (HD), conventional, and active sham transcranial direct current stimulation (n = 12).

Sensation	High definition	Conventional	Sham	χ^2^	*P*
Itching	1.0 (0.0–2.0)	1.0 (0.0–1.5)	1.5 (0.0–2.0)	0.261	0.878
Pain	0.0	0.0	0.0	N/A	N/A
Burning	0.0 (0.0–0.0)	0.0 (0.0–0.75)	0.0 (0.0–0.75)	1.0	0.607
Warmth/Heat	0.0 (0.0–1.0)	0.0 (0.0–0.75)	0.0 (0.0–1.0)	1.08	0.584
Pinching	1.0 (1.0–1.0)	1.5 (1.0–2.0)	1.0 (1.0–2.0)	0.75	0.687
Metallic/Iron taste	0.0 (0.0–0.0)	0.0 (0.0–0.0)	0.0 (0.0–0.0)	2.0	0.368
Fatigue	0.0	0.0	0.0	N/A	N/A
Beginning	1.0 (1.0–1.75)	1.0 (1.0–1.0)	1.0 (1.0–1.75)	0.5	0.779
Duration	2.0 (1.0–3.0)	3.0 (2.0–3.0)	2.0 (1.25–3.0)	3.56	0.169
Affect performance	0.0	0.0	0.0	N/A	N/A

Data described as median (interquartile range); N/A = not applicable.

#### Effect of tDCS on the outcome measures

**Figure 5:**
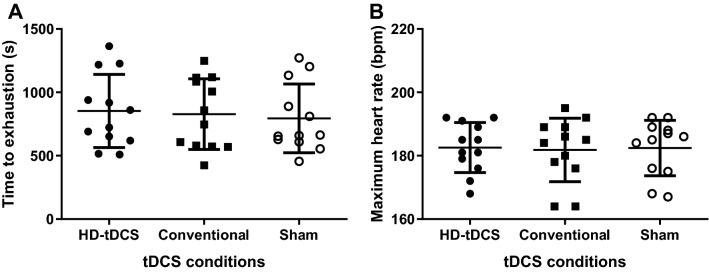
Effect of anodal high-definition, conventional, and sham tDCS on the time to exhaustion (**A**) and maximum heart rate (**B**) in time to exhaustion test with a constant load at 80% of peak power in athletes (n = 12). The data is presented as the mean, standard deviation, and individual data points.

Figure 5 displays the results in terms of TTE and maximum HR for each tDCS condition. There was no effect of tDCS condition in TTE [F_(2, 22)_ = 0.874; *P* = 0.43; η^2^p = 0.074; Fig. 5A]. Likewise, there was no difference in HR_max_ [F_(2, 22)_ = 0.329; *P* = 0.723; η^2^p = 0.029; Fig. 5B]. The effect size for TTE performance was g = 0.20 (95% CI − 0.60 to 1.00) for HD-tDCS vs sham, g = 0.12 (95% CI − 0.68 to 0.92) for conventional tDCS vs sham, and g = 0.09 for HD-tDCS vs conventional tDCS. An auxiliary analysis was performed to test if there was any difference in the TTE between the experimental conditions and the reproducibility phase. There was no difference in the TTE [F_(1.95; 21.4)_ = 1.537; *P* = 0.24; η^2^p = 0.123] between HD-tDCS (853.1 ± 288.6 s), simulated conventional (827.8 ± 278.7 s), sham (794.3 ± 271.2 s), and the TTE sessions performed to assess reproducibility of performance (TTE1 = 751.1 ± 261.6 s or TTE2 = 770.8 ± 250.6 s).

**Figure 6:**
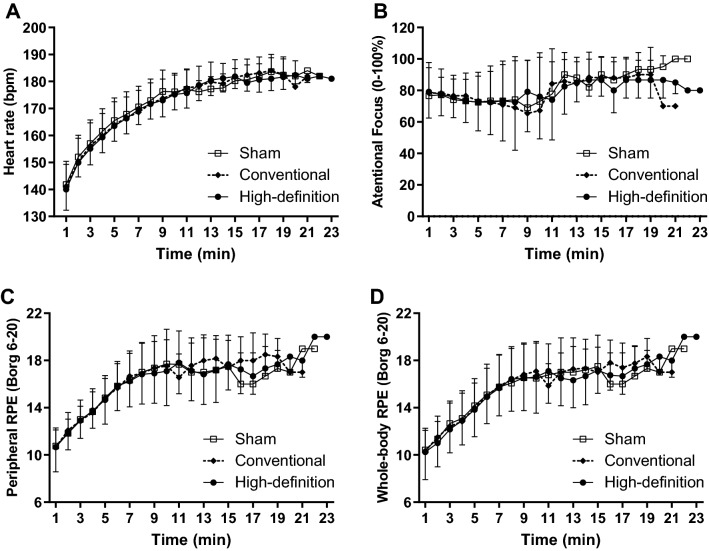
Effect of anodal high-definition, conventional, and sham tDCS on heart rate (**A**), associative thoughts (**B**), peripheral (**C**), and whole-body perceived exertion (**D**) during the time to exhaustion test with a constant load at 80% of peak power in athletes (n = 12). The data is presented as the mean and standard deviation.

Figure 6 summarizes the results of HR, associative thoughts, peripheral and whole-body RPE during TTE. There was only a significant main effect of time on the HR [F_(18, 18)_ = 61.10; *P* < 0.0001; η^2^p = 0.984]. There was no mains effect of condition [F_(2, 2)_ = 0.041; *P* = 0.960; η^2^p = 0.040] and condition × time interaction [F_(36, 36)_ = 0.766; *P* = 0.786; η^2^p = 0.468] (Fig. 6A). There was only a main effect of time on peripheral RPE [F_(16, 32)_ = 12.775; *P* < 0.0001; η^2^p = 0.865], with no effect of condition [F_(2, 4)_ = 0.409; *P* = 0.689; η^2^p = 0.170] or condition × time interaction [F_(32, 64)_ = 0.733; *P* = 0.830; η^2^p = 0.268] (Fig. 6C). Similarly, a main effect of time was found on whole-body RPE [F_(16, 32)_ = 12.775; *P* < 0.0001; η^2^p = 0.865], with no main effect of condition [F_(2, 4)_ = 0.409; *P* = 0.689; η^2^p = 0.170] or condition × time interaction [F_(32, 64)_ = 0.733; *P* = 0.830; η^2^p = 0.268] (Fig. 6D). No main effect of condition [F_(2, 2)_ = 1.00; *P* = 0.50; η^2^p = 0.50], time [F_(16, 16)_ = 1.00; *P* = 0.50; η^2^p = 0.50] or condition × time interaction [F_(32, 32)_ = 1.00; *P* = 0.50; η^2^p = 0.50] was found for associative thoughts (Fig. 6B).

There was no difference in baseline mood states between tDCS conditions: **vigor** [9.0 (5.5–11.75) vs. 9.0 (5.75–12.0) vs. 10.0 (6.0–12.0); χ^2^ = 4.42; *P* = 0.110], **fatigue** [0.5 (0.0–1.0) vs. 0.5 (0.0–1.0) vs. 0.0 (0.0–1.0); χ^2^ = 3.44; *P* = 0.179], **tension** [0.0 (0.0–0.75) vs. 0.0 (0.0–0.75) vs. 0.0 (0.0–0.75); χ^2^ = 0.200; *P* = 0.905], **confusion** [0.0 (0.0–0.0) vs. 0.0 (0.0–0.0) vs. 0.0 (0.0–0.0); χ^2^ = 2.00; *P* = 0.368], **anger** [0.0 (0.0–0.0) vs. 0.0 (0.0–0.0) vs. 0.0 (0.0–0.0); χ^2^ = 2.00; *P* = 0.368], and **depression** [0.0 (0.0–0.0) vs. 0.0 (0.0–0.0) vs. 0.0 (0.0–0.0); χ^2^ = 2.00; *P* = 0.368], for sham, simulated conventional, and HD-tDCS, respectively.

## Discussion

In the present study, we applied HD-tDCS and conventional tDCS in order to test the hypothesis that the improved focality and greater magnitude of neuronal excitability of HD-tDCS showed in the previous studies^10^ would improve the TTE in athletes to a greater extent than conventional tDCS. Briefly, we found no effect of either form of tDCS application on exercise performance, physiological, or perceptual responses to exhaustive exercise. The present results are in contrast with a recent meta-analysis by Machado et al.^5^ who found that a-tDCS over M1 improved cycling performance by ~ 93 s (95% CI = 27.39 s–159.43 s). However, this significant effect was strongly influenced by a single study. Our findings are contrary to previous studies that assessed the effect of tDCS on the exercise performance of athletes^16,35^. Okano et al.^16^ applied a-tDCS over the TC before incremental exercise in elite cyclists and found increased time to exhaustion and peak power compared to sham. Sasada et al.^35^ applied a-tDCS over M1 of collegiate athletes from various modalities (e.g. track and field, basketball, baseball, etc.) before an all-out 30-s sprint on a cycle ergometer and found an increased mean power compared to cathodal tDCS, but not compared to sham. Therefore, the findings on the effects of tDCS on athletes’ physical performance are still questionable.

Likewise, studies enrolling physically active subjects have also shown inconsistent findings^17,18,24^. While Vitor-Costa et al.^17^ and Angius et al.^18^ found increased TTE after a-tDCS over M1, Angius et al.^24^ did not. However, it is noteworthy that, despite it is largely diffused that only an effect of at least moderate size is relevant, the importance of an effect is ultimately determined by the context it is applied^54^. In the case of athletes, even a small effect size can have an important impact on the outcome of a competition. For instance, it has been demonstrated that a difference of less than 1% on the average speed of intense endurance events lasting ~ 45 s to 8 min such as cycling, running, swimming, kayak, and rowing would have changed podium positions in the London 2012 and Rio 2016 Olympics games^55^. Hence, if a reliable tDCS-induced performance enhancement it might represent an interesting advantage in the “real-world”, for athletes in competition. However, at present, it remains speculative.

In this regard, one should consider the difference and reproducibility in performance between athletes and non-athletes individuals. Athletes are accustomed to performing the exercise of maximum nature almost daily and this makes them more likely to present reproducible maximal performances. Additionally, considering their high fitness level and competitive interest, in maximal effort exercise, athletes are more eager to perform the exercise. In fact, in the present study, the outcomes measures were reproducible within the two sessions, with the main outcome, TTE time showing excellent reliability (ICC = 0.938) and a relatively low MDC (14.7 s). On the other hand, non-athlete individuals (e.g., physically active) might be more susceptible to suffer interference from different sources, such as emotional variations or even fear to exercise at exhaustive intensities, which increases performance variability, and, therefore, more probable their performance to be influenced by tDCS or other interventions. One possible explanation for the present results is that a ceiling effect in athletes’ physical performance may exist, which may be an explanation for the null findings in the present study. In fact, the baseline level of function has been shown to influence both cognitive and motor effects of tDCS^56^, so that individuals with a poorer baseline level of function improves after tDCS while those with higher levels of function display lower or no improvements or even a detrimental effect on performance. For instance, Furuyama et al.^57^ showed that anodal tDCS over M1 improved fine motor control in musically untrained individuals, but decreased the performance of experienced pianists. Similarly, Rosen et al.^58^ showed that anodal tDCS over the right dorsolateral prefrontal cortex enhanced improvisation performance in jazz pianists with less experience but worsened improvisation performance in those with more expertise. Therefore, it is likely that less trained individuals may likely benefit from a higher degree of the possible performance enhancement effect of tDCS compared to athletes.

Nonetheless, Pollastri et al.^46^ recently demonstrated a 1.3% faster 15 km time-trial in eight elite cyclists after bilateral HD-tDCS over the DLPFC (F3 and F4) with 1.5 mA for 20 min. As previously mentioned, the performance improvement shown by Pollastri et al.^46^, despite seeming small may represent an important advantage considering the high level of performance of the sample^55^. The difference between the present study and the study by Pollastri et al.^46^ may be explained by the different areas of stimulation, as HD-tDCS was applied over M1 in the present study and over the DLPFC in their study. In fact, another recent study found an effect of conventional tDCS over M1 (2 mA for 20 min) on mood but not on performance in an 800 m swimming test in elite triathletes^29^. Furthermore, different forms of exercise performance measures were used. In the present study, we used an open-loop task (i.e., TTE) defined by the absence of a known endpoint (i.e., individuals cycle for as long as possible). While in the Pollastri study they used a closed-loop exercise (i.e., time trial), defined by the existence of a known endpoint (i.e., individuals cycle for a fixed distance or duration). Future studies should compare the effect of tDCS over different areas of stimulation (e.g., DLPFC vs. M1) and also using different exercise test protocols (e.g., TTE vs. time-trial).

Studies have looked into the factors that may explain interindividual variability^56,59,60^**.** Wiethoff et al.^60^ showed that about 50% of the participants had minor or no change in cortical excitability after tDCS. From the participants who responded to tDCS, 36% presented the “classical” polarity-dependent response in cortical excitability (anodal-excite/cathodal-inhibit), while 21% displayed the inverted polarity-dependent response (anode-inhibit/cathode-excite)^60^. In addition, both polarities were excitatory or inhibitory for 38% and 5% of the participants, respectively^60^. The factors that might influence individual responses to tDCS include anatomical variations, the organization of local circuit, the basal level of function, psychological state, level of neurotransmitter and receptor sensibility, baseline neurophysiological state, and even genetic aspects^56^. Future investigations should focus on possible predictors of the tDCS-induced modulation in exercise performance.

The baseline psychological state was not different between experimental conditions. In this regard, Marcora^61^ proposed in his psychobiological model that exercises tolerance is influenced by how much effort an individual is willing to exert, and the continuation of exercise is perceived as impossible. In addition, Beedie, Terry, and Lane^62^ in a meta-analysis design to investigate the effects of mood states on athletic achievement and exercise performance showed that the domains of mood state influence the exercise performance for different types of sports activities (e.g. open-loop, closed-loop, short, long, team), especially, the domains of perceived vigor, confusion, and depression. Therefore, this result confirms that exercise performance variations were not due to oscillations in psychological states.

Similar to performance, a-tDCS did not change HR, attentional focus (i.e. associative thoughts), peripheral, and whole-body RPE. This result is also in line with previous studies that did not find a significant effect of a-tDCS over the motor representation of the lower limbs on physiological or perceptual responses^24^, even in the presence of improved exercise performance^17,30^. Only Angius et al.^18^ found decreased RPE after applying a-tDCS over the motor representation of the lower limbs, but with unchanged HR. One possible explanation for the lack of change in the physiological or perceptual responses in the present study may be since the stimulated area has no clear relationship with neither cardiac autonomic control nor the processing of sensorial or cognitive information, such as the prefrontal or insular cortex, as the target for tDCS in other studies^16,63^.

The lack of significant performance improvement could be partially explained by two main factors, one related to the sample size and the other related to the sham method used. First, we calculated the sample size based on the effect size of a previous study with physically active individuals^17^. However, as discussed above non-athletes are more likely to have effects of greater magnitude. Therefore, even though we have decreased the effect size for the sample size calculation it may have overestimated the effect of tDCS on exercise performance. In fact, in a recent meta-analysis^5^ we have found that a significant effect of a-tDCS on performance in the TTE test, however, Vitor-Costa et al.^17^ study presented a disproportionate weight, which influenced the meta-analytic result.

Regarding the sham method, we adopted an active sham approach for better blinding of the participants^64^. This method was chosen because (1) the commonly used method where current is ramped up and 30 s after it is ramped down^65^, with no current for the remaining time, has been consistently shown to be inadequate to deceive participants^64^ and (2) the sensations from HD-tDCS is stronger than conventional and lasts longer, thus, with the active sham method the blinding of participants related to tDCS condition is improved^48–50^. In fact, no difference was found between sensations related to the HD-tDCS, conventional, or sham tDCS (see Table 2), confirming that the sham method was efficient for blind participants. However, even though the electrical current flows from one electrode to another closest to it and, in this way, most of the current is shunted on the scalp and skull, some current does penetrate the cerebral cortex and a possible modulation from this active sham method may not be ruled out^49^. Moreover, expectations, placebo, and nocebo effects of tDCS have been reported^66,67^. Rabipour et al.^66^ found greater improvements in working memory after tDCS subsequently to priming with high (i.e., tDCS is effective) compared to low expectations (i.e., tDCS is of uncertain effectiveness). Participants receiving active tDCS with low expectation priming showed the lowest performance^66^. Additionally, sham tDCS with either positive or negative priming of tDCS effects induced placebo and nocebo effects on reward-based learning performance^67^. However, in the present study, when compared to the two TTE performances in the reproducibility phase (i.e., used as control sessions as no intervention was performed), there was no difference among any of the experimental conditions (HD-tDCS, conventional tDCS, or sham) and two TTE tests of the reproducibility phase. This finding supports the idea that any experimental condition induced performance enhancement.

Our trial adapted a conventional tDCS montage previously reported to increase exercise performance in the TTE test in a cycle simulator with 80% of peak power^17^. Since “return” electrodes cannot be considered inert^39^, we positioned conventional tDCS return electrode to avoid confounds from current flow to non-predicted anterior brain areas (e.g., premotor cortex, supplementary motor area, prefrontal region). All the montages tested here were supported by high-resolution computational models of brain current flow, moreover with experimental attention to ensuring that similar head-gear and preparation techniques across montages did not compromise blinding. Because tDCS outcomes are montage and intensity specific, our results may be distinct from those with a different dose, even nominally targeting the same motor regions^5,15,17,18^.

From a practical perspective, taken together the findings of the present study and the available literature does not give clear support to the idea that tDCS (at least using a single session) can improve physical performance in athletes, and, therefore, the spreading use by and/or commercialization of tDCS for physical performance improvement to the open community is not based on evidence. More controlled studies in this field are certainly warranted. In this regard, some strengths of the present study include: (a) the use of computational modeling to predict electrode placement, current intensity, and density to reach the target area, (b) the use of different tDCS application technique, namely HD-tDCS, (c) the assessment of a sample of well-conditioned athletes. Our main limitation was the low achieved power due to the small sample size. However, despite the fact we assessed a low sample size, the number of subjects was determined a priori based on a previous study with a similar design. In addition, the assessment of athletes is particularly difficult considering that for participating in the study they should refrain from exercise training for at least 24 h before the assessment session, it implies in at least four training sessions lost to participate in the study. This was the main reason for not taking part in the study and/or for abandoning it. Moreover, the lack of measures of neurophysiological responses following tDCS (i.e., corticospinal and corticocortical excitability, brain activity, etc.), differences between HD-tDCS and the simulated conventional tDCS, and exercise protocol may also be considered as limitations.

## Conclusions

In the present study, there was no significant effect of a single session of either HD-tDCS or conventional tDCS on exercise performance and psychophysiological responses in athletes. Future studies may test whether a ceiling effect exists in athletes compared to non-athletes. Additionally, the effect of tDCS in other brain areas, such as the DLPFC, may provide different results and should be tested by future studies. Finally, the predictors of tDCS effects and factors associated should be targeted in the exercise science field in order to understand whether tDCS works, to whom, and under which conditions.

## Data Availability

The data regarding the present study are available from the corresponding author on reasonable request.
